# Supplementary figures and images for: Identification of Circular RNAs of Testis and Caput Epididymis and Prediction of Their Potential Functional Roles in Donkeys
Source: Genes (Basel). 2022 Dec 25;14(1):66. doi: 10.3390/genes14010066 (PMC9858477; doi:10.3390/genes14010066)

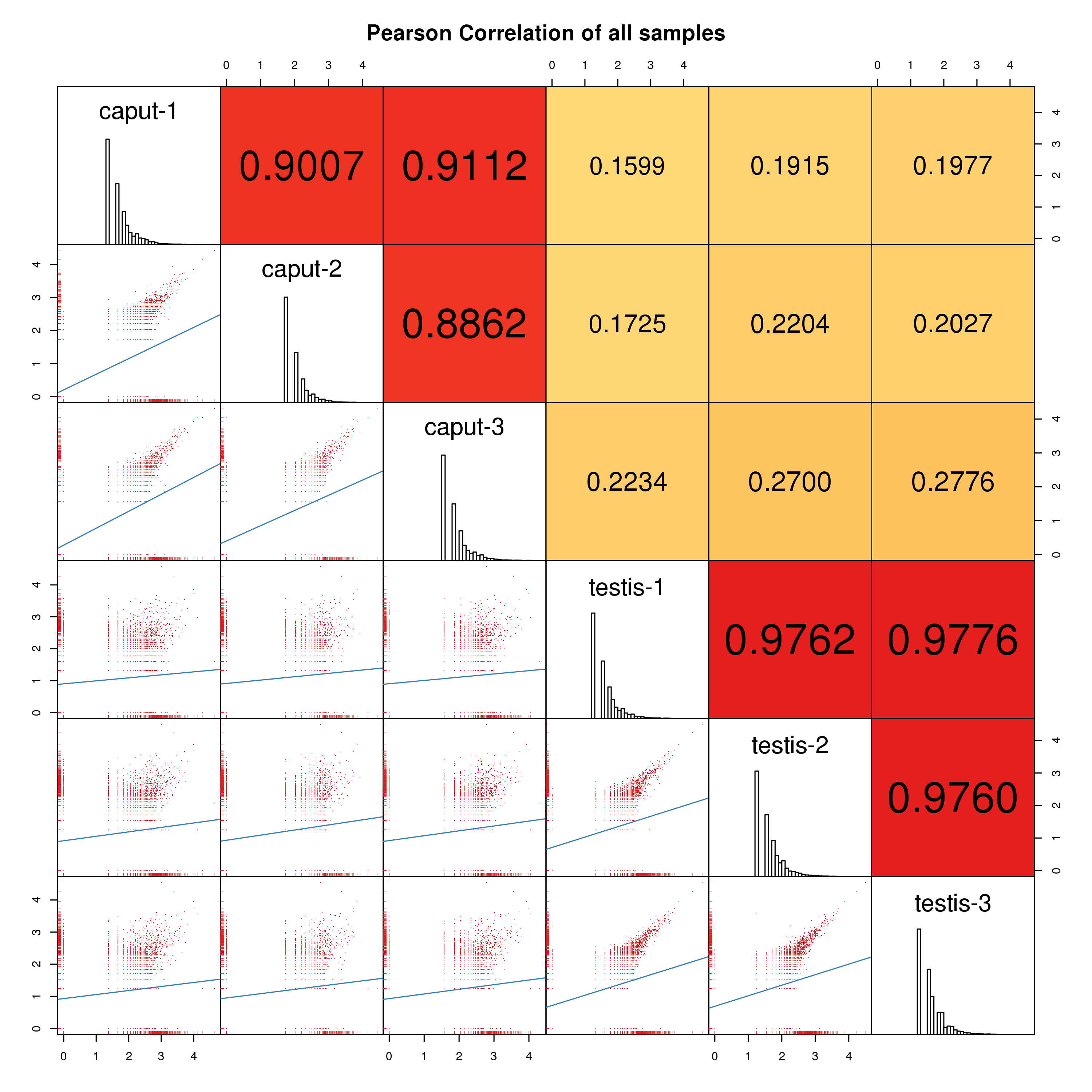

Supplement: Supplementary file 1 [file genes-14-00066-s001.zip › Supplementary Figure S1.png]
